# From theory to practice: modeling performance in breaking

**DOI:** 10.3389/fspor.2024.1489456

**Published:** 2024-12-17

**Authors:** Lucie Lerebourg, Brice Guignard

**Affiliations:** ^1^ Centre de Ressources, d’Expertise et de Performance Sportives des Pays de la Loire (CREPS PDL), La-Chapelle-sur-Erdre, France; ^2^ Univ. Rouen-Normandie, Laboratoire Centre d’Études des Transformations des Activités Physiques et Sportives (CETAPS—UR 3832), Mont-Saint-Aignan, France; ^3^ Universite Claude Bernard Lyon 1, LIBM, Interuniversity Laboratory of Human Movement Sciences, UR 7424, Lyon, France

**Keywords:** breakdance, sport, hip-hop, urban dance, optimization, Olympics

## 1 Introduction

The introduction of a hip-hop dance event, breaking, at the Youth Olympic Games (YOG) in Buenos Aires in 2018, inevitably marked a turning point for hip-hop culture and its dancers (1). Breaking was a real success at the YOG (e.g., first inclusion in an Olympic event, worldwide visibility, introduction of an objective evaluation system for the battles, strengthening of breaking institutions, inspiration for a new generation) (2), and the Paris 2024 Organizing Committee subsequently proposed it to the International Olympic Committee (IOC) as an additional discipline to Summer Olympics in Paris 2024 (1). Following this decision, and in the interests of transparency, fairness and objectivity (i.e., to avoid any controversy linked to the votes), a Judging System (i.e., scoring) designed and tested during the YOG with a more elaborate codification (3, 4) than the show of hands was introduced in major breaking competitions in order to qualify the dancers' performance (5).

Performance in breaking, as in other sports, is multifactorial (6–8). It is achieved when the athlete's physical abilities align with the demands of the task. The rules governing the competition (3) shaped by the evolution of the sport define the conditions under which athletes compete and determine victory. These rules not only structure the performance but also make the sport accessible to a broader range of participants, including different categories of hip-hop/urban dancers (e.g., age, gender). Breaking, an urban dance style that originated in the Bronx, New York in the 1970s, has been shaped by these evolving standards to become a competitive discipline (4, 9–11). Indeed, whatever the level of execution of the action, as soon as the relationship between the athlete's physical capacities and the sporting task to be accomplished is optimized, the notion of performance can be apprehended. The conditions and standards according to which athletes will compete and perform are dictated by the sporting rules (3). The rules, which are both the basis and the fruit of the evolution of a sporting practice, make it possible to structure performance and determine victory, but also to adapt the conditions under which an event or discipline is practiced making them accessible to different hip-hop/urban dancers, such as breaking (e.g., age categories, gender). Breaking is an urban dance style that originated in the Bronx borough of New York City in the mid-1970s (9–11). Breaking has evolved into a global cultural art form with many elements of sport, given its athletic nature (4). At present, breaking performance, in federal events, is defined in terms of the ranking required to qualify for major championships such as the European, the World Championships or the Olympic Games (OG). Any result obtained in an official competition enables competitors to earn ranking points and appear in rankings at various levels (3). Levels of performance can be assessed based on ranking, correlated with the results obtained in the various battles (3).

To have an impact on performance, it seems essential to identify the factors that define it, and which are grouped together in the form of a model commonly referred to as the “performance model” (12). Beyond a systemic approach that leads us to view performance through a multi-factorial and dynamic prism, the approach needs to be complemented with an analytical eye to analyse the multiple relationships, the cause-and-effect links that interact to build performance.

Due to its rapid institutionalization to reach the standard of the IOC, the aim of this paper is to attempt to model performance in breaking, an additional discipline at the Paris 2024 OG (3, 13). As the scientific literature based specifically on this cultural art form with many elements of sport is relatively limited, the contribution of a more objective analysis to breaking performance linked to a judging system (3, 4), in the same way as other sports disciplines judged by humans such as gymnastics, figure skating, skateboarding, etc., appears to be an interesting prospect for work.

## 2 The current landscape

### 2.1 The internal logic of breaking

The internal logic of a sport or artistic physical activity refers to “the identity card of the activity in question, which brings together its most salient relevant characteristics” (14). Each sport has its own internal technical and tactical logic that determines the required effort for the athletes (15, 16) to perform.

In breaking, the competition relies on “question & answer” battle in duos or in crews performed during successive rounds where the effort is intermittent: while one dancer performs, the other rests before switching roles. The duration of both effort and recovery is variable, depending on the dancers (16, 17). Dancers are named break-boy or b-boy and break-girl or b-girl, as a function of sex (3, 11). The event is codified and rooted in choreographic foundations, with the presence of a disk jockey (3, 4), a Master of Ceremonies (i.e., the event host, and an audience). In individual battles (1 vs. 1), on strategy could involve analyzing the opponent's strengths and weaknesses (physical, artistic, or interpretive qualities) and adapting each round to gain an advantage and win over the judges (4). Dancers must also adapt to the DJ's music, which is unknown in advance (13).

As with any duel-based competition, it is essential to establish a strategy and carry out tactical work (e.g., specific placement work to manage opponent, energy, fatigue or injury…) (16, 18). Tactical work can be related to the occupation of space (i.e., circle or cypher), to orientations and to the stage play, for example (11). In fact, in a battle, the dancer expresses her/himself above all in front of an opponent who must be destabilized, in front of a jury that judges her/him (i.e., the jury perform the ranking), but also in front of an audience that, depending on its reaction, may become involved in the fight (3). From a strategic point of view, as the practice of breaking has been democratized, attracting more and more dancers, organizers are now planning “Pre-Selection” (i.e., Phase 1*,* first stage of a World Dance Sport Federation, WDSF, breaking event), considered to be an “antechamber” to the competition that should not be neglected from a strategic point of view. After Pre-Selection, the competition continues with various phases including “Pre-Qualifier”, (i.e., TOP 64 and TOP 32), Round Robin (i.e., TOP 16, the competitors are assigned into 4 Groups), and the Knock-Out Phase (i.e., quarter-final, semi-final, battle for third place, and the final) (3, 11, 16, 17). In all cases, whatever the phase, the goal is to win the rounds by getting as many votes as possible from the judges, adapting and destabilizing the opponents throughout the competition to go as far as possible. The opponent often uses “Bronx Rocking” gestures (11) to destabilize by highlighting repetition, mistakes, or lack of control in a movement (11).

In breaking, the b-girl/b-boy uses different categories of fundamental movements called foundations, i.e., toprock, downrock, and freeze, to build their rounds like a construction game. Although this list is not exhaustive, breakers commonly start with toprocks (i.e., standing moves) to set the tone (16), then transition to downrocks, which include footwork (a variety of ground-based dance steps), power moves (complex, spinning movements requiring control over gravity and velocity), and acrobatics (aerial moves without ground contact, sometimes resembling gymnastics). A round often ends with a freeze, a pose in which the dancer stops moving entirely, creating a striking “photographic” impression for the spectator (3, 11, 16). These different components of dance therefore refer to cognitive, motor and affective qualities that need to be understood to optimize sport performance in breaking (5, 19).

### 2.2 Criteria for the evaluation of the judging system

**Figure 1:**
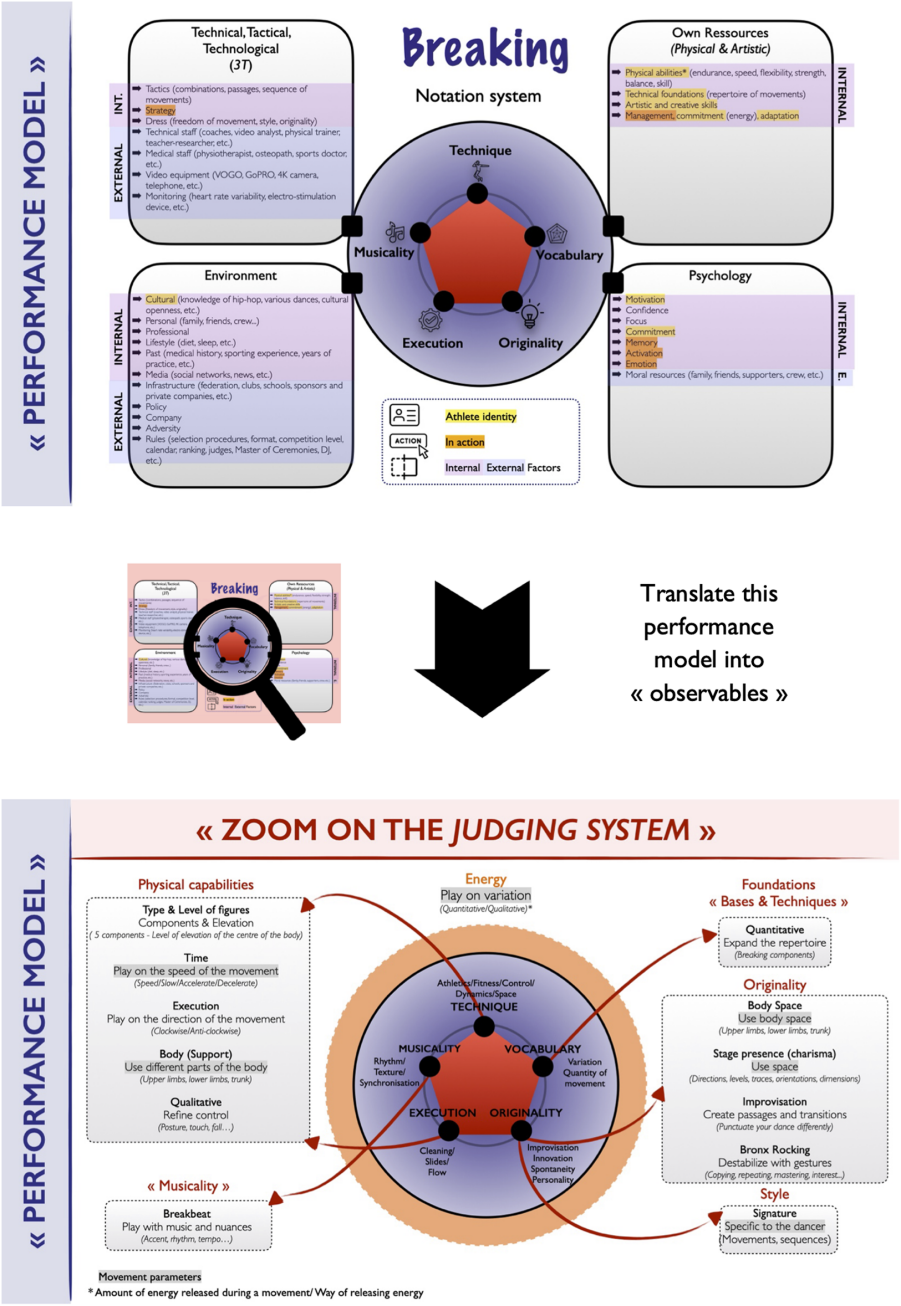
Illustration of a performance model of breaking. Top panel: integration of numerous “physiological and artistic”, “psychological”, “environmental” and “technical, tactical and technological” factors. Bottom panel: transcription of the performance model into “observables” in line with the current WDSF breaking judging system (Level A) that incorporates five criteria (technique, vocabulary, originality, execution, and musicality, as defined in Figure 1) (3, 4). Note 1: taking physical qualities as an example, the proposed breaking performance model considers several qualities such as: Endurance: this promotes motor efficiency, while at the same time appealing to the psychological aspect through the repetition of effort, as well as recovery; balance: this helps to improve efficiency and control of movement (rhythm and amplitude) and changes of direction; strength: coordinates and transfers power between the upper and lower limbs; speed: this is kinetic speed of movement, explosiveness and analytical movement frequency; skill/coordination: this contributes to a better understanding of the body’s schema; flexibility: the ability to mobilize a joint and its muscle groups to the greatest extent possible, with the best possible mobility. It facilitates development and progress in building up a repertoire of movements. Note 2: the technique criteria demonstrates a more fundamental understanding of the physiological control and dynamics required for clean execution of the moveset [major focus areas: athleticism, form (lines, angles, shapes), body control, dynamics, spatial awareness]. The vocabulary criteria demonstrates a more extensive range of moves than the opponent, acquiring and internalising an extensive, varied movement vocabulary, with minimal repetition of moves or movement patterns (major focus areas: variation, quantity of moves, repeat); the execution criteria demonstrates a greater ability to execute the moves clearly and with a high degree of cleanliness, minimising slips, falls or crashes (major focus areas: cleanliness, minimal to no Slips, crashes, or falls, consistency of flow, composition, storytelling); the musicality criteria demonstrates a greater ability to synchronise movements effectively with the music to adapt and respond more quickly to the rhythms and textures present in the music, and to anticipate moments to accentuate the performance (major focus areas: rhythm, texture, synchronicity, accenting); The originality criteria demonstrates a clearer ability to develop creative variations on basic movements, whilst showcasing one's own unique set of movements (major focus areas: improvisation, innovation, spontaneity, personality, response) (3, 4).

In the interests of transparency and fairness, notably with the arrival in the 2024 OG in Paris, scoring systems with a more elaborate codification than the show of hands (to determine the winner) have appeared fairly recently in high-profile competitions in order to avoid any controversy linked to the votes (3–5). Indeed, the WDSF has developed two levels of Judging System known as “The WDSF Breaking Judging System” (3, 4). Level A, currently defined by five criteria (Technique, Vocabulary, Originality, Execution, and Musicality, see Figure 1). This is adapted from the YOG in 2018. Level B, corresponding to a Three-fold system of only three broader categories (Physical, Artistic and Performative) (3, 4). Judges use a digital tablet to vote by adjusting a slider in real-time, always comparing breakers to their opponent rather than using an absolute scale (3). The interface adapts to the competition level, Level A or B, with approval from the WDSF Sports Department, in consultation with the organizer and the technical delegate (3).

### 2.3 Towards a performance model in breaking

Even before designing a performance model for breaking, it may be interesting to carry out a mapping of the activity and to take into account different aspects such as the resources specific to the activity (e.g., physical and artistic resources), the psychological resources, the environment (i.e., the context) in which the dancer is evolving, as well as the technical, tactical and technological resources (3, 4, 6, 12, 16, 17, 20, 21) (Figure 1).

In addition, there are also internal (i.e., dependent on the athlete and training) and external factors (i.e., independent and beyond the control of the athlete and coach, e.g., judges, DJ, opponent) which revolve around breaking performance, itself defined by criteria (i.e., technique, vocabulary, originality, execution, and musicality) grouped together in an evaluation system during WDSF breaking events (3). In the context of performance analysis, it seems interesting to think about the elements (i.e., metrics) that can be observed and quantified in breaking with the aim of optimizing sports performance. For each assessment criteria, several metrics have been defined to try to qualify the performance in breaking (Figure 1) like for example “type and level of figures” (components and elevation) for the technique criteria, “quantitative” (extensive range) for the vocabulary *(*i.e., repertoire of figures), “signature” (specific movements or sequences to the dancer) for the originality, and “breakbeat” (play with music and nuances) for the musicality criteria (Figure 1).

Thus, modelling breaking performance therefore needs to be broken down into elements that provide a better understanding of the different factors that need to be addressed (Figure 1). This model is based on the WDSF Breaking Rules and Regulations Manual (3), which itself draws upon foundational principles established by the Dance Adjudication Network (DAN) to ensure a structured, transparent, and multifaceted approach to judging breaking performances (4). Breaking, like other hip-hop dances, appears to be a specific activity, borrowing as many codes from art as from sport (1, 22). Considering these elements, the concepts of art, sport and, in this case, dance should be combined.

## 3 Discussion

Having studied the internal logic of this dance (16), considering its rules (3), its technical and tactical characteristics (3, 13, 17, 21, 23, 24), the type of music (5, 11) and the various elements that make it up (1), it is now easier to understand the breaking performance and to propose a performance model specific to this discipline.

The aim of this opinion was to design and propose a breaking performance model that would correspond to the Judging System criteria established by the WDSF and defining performance (3). In relation to the proposed performance model, it is noted that there are not as many observables that can be qualified and quantified for each assessment. In a sporting and artistic discipline such as dance (22), it is not always obvious or relevant to quantify all the parameters as observables. For example, it is potentially more difficult to assess the musicality (rhythm, accent) of the dancer than the technique, such as the quality of execution of the dancer's movements. However, this work on thinking about and modelling breaking performance should help us to understand this additional sporting and artistic discipline at the 2024 Summer Olympic Games in Paris (13), so that we can open up new perspectives of work in areas such as notation/video analysis (e.g., kinematic analysis, video coding/sequencing…) for example, to manage strategy in relation to the opponent, fatigue, the risk of injury, in an objective of optimizing performance.
